# Metformin, the Rise of a New Medical Therapy for Endometriosis? A Systematic Review of the Literature

**DOI:** 10.3389/fmed.2021.581311

**Published:** 2021-05-11

**Authors:** Emanuela Stochino-Loi, Attila L. Major, Tessa E. R. Gillon, Jean-Marc Ayoubi, Anis Feki, Jean Bouquet de Joliniere

**Affiliations:** ^1^Department of Obstetrics and Gynecology, Cantonal Hospital, University of Fribourg, Fribourg, Switzerland; ^2^Femina Gynecology Center, Geneva, Switzerland; ^3^Department of Obstetrics and Gynecology, Foch Hospital, University of West Paris, Suresnes, France

**Keywords:** endometriosis, medical therapy, metformin, contraceptives, fertility

## Abstract

Medical treatments for endometriosis aim to control pain symptoms and stop progression of endometriotic lesions. However, their adverse effects and their contraceptive effect in women who desire pregnancy, limit their long terms use. Although there is only one study investigating the effects of metformin on women with endometriosis, metformin seems to have a unique therapeutic potential. It may be a helpful anti-inflammatory and antiproliferative agent in the treatment of endometriosis. As such metformin may be more beneficial thanks to the lack of serious side effects.

## Introduction

Endometriosis affects up to 15% of women of reproductive age (1). It is characterized by the presence and/or growth of endometrial tissue (both epithelial and stromal cells) outside the uterine cavity causing chronic inflammation inside and/or outside the pelvis. Thereby, in rare cases endometriosis can also appear in post-menopause (2). In addition to the peripheral estrogen production, a high circulating level of estrogen can be induced by an external source, especially in the form of phytoestrogens and HRT. Phytoestrogens exert estrogenic effects on the uterus, breast, and pituitary and could also support the growth of endometriotic lesions (3–6).

Interleukin-6 may play a role in the pathogenesis of endometriosis by initiating or sustaining the inflammatory response in the pelvic fluid (7).

Increased local production of estrogen in endometriotic tissue was suggested to be important for the growth of the lesion (8).

Endometriosis may be asymptomatic, however it frequently causes infertility and pain, such as dysmenorrhea, dyspareunia, chronic pelvic pain, dysuria and dyschezia (9).

Combined hormonal contraceptives, are recommended as first-line therapy followed by analogs and antagonists of GnRh. In case of failure of the initial treatment, recurrence, or multiorgan involvement, a multidisciplinary surgery is recommended (10).

Medical treatments of endometriosis are suppressive, not curative, and symptoms can continue after medical treatment or conservative surgery. These treatments present limitations related to side effects such as a contraceptive action for women who desire pregnancy, costs and resistance to progesterone (11).

Progesterone plays an important role in decreasing the inflammatory response. But adversely chronic inflammation can induce a progesterone-resistant state. A consequence of impaired progesterone action is that hormonal therapy is rendered ineffective for a subset of women with endometriosis (12). Synthetic progestins, such as dienogest, or high dose depot of progestins may overcome this phenomenon by modifying progesterone receptor expression and decreasing proinflammatory cytokines (13). However, they can be associated with strong side effects and therefore not be well-tolerated (14).

For these reasons, finding an alternative treatment to associate to a hormonal treatment or to propose as mono-therapy in patients where hormonal treatment is either contraindicated, not well-tolerated or not adequate (e.g., desired pregnancy), should be a priority.

Metformin is a widely used anti-diabetic agent that improves insulin sensitivity and is generally well-tolerated. Results of numerous clinical studies indicate that metformin use is associated not only with decreased incidence of cancer in diabetic population, but also with the better outcome in cancer patients (15). It is already used for improving symptoms of polycystic ovary syndrome (PCOS) (16).

Mansfield et al. (17), Isoda et al. (18) and Takemura et al. (19) all demonstrated that Metformin reduces inflammation. Takemura et al. (19) additionally described the inhibition of aromatase and proliferation in human endometriotic stromal cells in cell culture after adding metformin. Endometriosis induces a chronic proinflammatory state which itself is a main factor causing progesterone resistance.

Metformin may be the missing link for a successful treatment of patients with endometriosis, avoiding important side-effects.

## Materials and Methods

A search of the Medline/PubMed and Embase databases was performed to identify all published English language studies on endometriosis and metformin.

Keywords such as “Metformin and Endometriosis”, “new endometriosis treatments,” “rat model and endometriosis,” “side effects endometriosis” were used.

## Results

Six articles about the effect of metformin and endometriosis were found: two studies used rat models, three *in vitro* models and only one study was performed in patients with a diagnosis of endometriosis.

### Experimental Study *in vitro*: Is Metformin Effective in Endometriosis Disease Treatment?

The first *in vitro* study, published in 2007, aimed to determine if metformin may be effective for the treatment of endometriosis (19). It evaluated the effects of Metformin on inflammatory response, estradiol production and proliferation of endometriotic stromal cells. In detail, the study showed that endometriotic cells produced IL-1-Beta that induced IL-8 secretion that promotes endometriotic cells' proliferation. Metformin suppressed IL-1Beta production, IL-8 inhibition production and stopped proliferation of endometriotic cells.

The second *in vitro* study (20) suggested that metformin could inhibit prostaglandin E2 (PGE2) by suppressing the expression of CYP19A1 and inhibiting the aromatase activity in endometriotic cells. Aromatase induced estrogen synthesis leads to the growth of the endometrial implants, COX expression, prostaglandin secretion, which further induces aromatase activity. Unlike GnRH agonists, aromatase inhibitors block estrogen synthesis both in the periphery and the ovaries. This mechanism is particularly helpful in postmenopausal women with endometriosis where peripheral fat is the predominant source of estrogen (21). Aromatase P450 is expressed in both the eutopic and ectopic endometrium of patients with endometriosis while it is not detectable in the eutopic endometrium obtained from healthy women and in endometriosis-free peritoneal tissue (22). These observations prompted several investigators to inhibit this enzyme by using third-generation non-steroidal (type II) aromatase inihibitors (AIs), such as AZT and letrozole, in order to treat endometriosis. AIs are not licensed for the treatment of endometriosis and they may only be considered in a research environment when all other options have been exhausted (23). Moreover, studies investigating letrozole and ATZ for the treatment of endometriosis employed the dose established for the treatment of breast cancer. Importantly, a lower dose of AIs may sufficiently inhibit the activity of peripheral aromataseP450 in patients with endometriosis. The administration of AIs to women of reproductive age is associated with several side effects, including hot flashes, weight gain, bone and joint pain, muscle aches, and less frequently mood swings, headache, vaginal spotting, fatigue, dizziness, depression, increase appetite, insomnia, rash, and decreased libido (24).

In an article published in 2015 (25), ectopic endometriotic stromal cells (ESC) expressed and secreted higher Wnt2 protein compared with normal endometrial stromal cells (NSC). Metformin decreased the expression and secretion of Wnt2 in ESC. Wnt2/β-catenin signaling was involved in stromal-epithelial cells interaction in endometriosis and promoting their growth. Metformin might regulate the stroma-epithelium communication via Wnt2-mediated signaling in endometriosis (25).

These findings *in vitro* suggest the unique therapeutic potential of metformin, as an effective treatment of endometriosis, and the importance of future investigations.

### Experimental Studies in Rat Models: Is Metformin Effective in Endometriotic Disease Treatment?

The study of Yilmaz et al. (26) is the first study that demonstrated that after inducing endometriotic lesions in rats, Metformin effectively causes regression of endometriotic implants and decreases the level of VEGF and MMP-9 (metalloproteinase 9), interleukin-1beta, interleukin-8. The inactivation of aromatase causes regression of the size and atrophy of the endometriotic implants.

The study of Oner et al. (27) compared Letrozole to Metformin. Both groups showed a regression of endometriotic implants, but in the group treated with metformin there was an additional reduction of adhesions.

### Is Metformin Effective in Clinical Practice?

Only one clinical study was done (28) on endometriosis and metformin. The study included 90 patients with endometriosis subdivided in to 2 groups: the control group (CG) had no treatment and the active group (AG) was treated with Metformin only.

Only ten patients from the AG refused to continue to be treated with metformin due to gastrointestinal problems.

The AG included finally 35 infertile patients, whereas the CG included 34 cases. In both groups patients consisted of stage 1–2 endometriosis diagnosed by laparoscopy and were complaining of one or more symptoms such as pelvic pain, dyspareunia or menorrhagia.

Patients in the AG were given 500 mg of oral metformin, three times a day for 6 months.

The cytokines levels of VEGF, IL-6 and IL-8 were evaluated at the start of the study and again after 3 and 6 months.

In the AG, pelvic pain decreased from 25.7 to16.1% after 3 months and to 7.69 % after 6 months; dysmenorrhea decreased from 51.4 to 32,2% after 3 months and to 23% after 6 months; and dyspareunia decreased from 20 to 16.1% after 3 months and to 7,7% after 6 months. The pregnancy rate of the AG group increased at the same time from 0 to 11.4% after 3 months and to 25.7% after 6 months. The results show that the AG (treated with Metformin) presented a reduction of dysmenorrhea, a reduction of pelvic pain and dyspareunia. Furthermore, the percentage of pregnancy after 6 months of metformin therapy was significantly higher when compared to the CG or a treatment period of only 3 months.

### Can Endometriosis Be Compared to Cancer?

Although it is not a malignant or premalignant disorder (only 0.5–1% of endometriosis are complicated by neoplasia (29), endometriosis exhibits similar mechanisms as cancer such as proliferation, migration, neo-angiogenesis, and recrudescence (30, 31).

For this reason, it is interesting to evaluate the mechanism behind the development of endometrial cancer because it is the result of disruption of the balance between estrogen-stimulated growth and progesterone-induced growth modulation.

Metformin has been studied in patients with endometrial cancer and the results show that metformin abrogated the effects of E2 on cell proliferation. Metformin has multifaced benefits: metformin exerts its anti-cancer effects by decreasing incidence of different cancers and inhibition of proliferation and migration of cancer cells, activation of apoptosis, and reducing epithelial mesenchymal transition (EMT) and metastasis (32). In cancer cells, the ability of metformin to alter cancer metabolism and mitochondrial function and to modulate intracellular signaling activity related to key oncogenic pathways such as the Ras/Raf/MEK/ERK, PI3K/Akt, and mTOR pathways, retards cancer cell growth, proliferation, migration, increases cell death, and inhibits EMT, invasion, and metastasis. While the activation of AMPK seems to play a key role to the many of the beneficial anti-cancer effects of metformin, AMPK independent effects have also been reported. However, most of the mechanistic data on the anti-cancer effects of metformin were derived from *in vitro* experiments using cancer cell lines and thus may not reflect the mode of action of metformin in an *in vivo* or clinical setting (33–39).

However, Metformin significantly decreased estrogen receptors but did not consistently affect the expression of progesterone receptors (40). This is why a treatment combining Metformin with hormonal treatments may be a good option to try.

### Metformin Related to PCOS and Inflammation

Metformin is a widely used anti-diabetic agent, generally well-tolerated, that improves insulin sensitivity and is used for the treatment of polycystic ovary syndrome (PCOS) (16, 41). Some of the effect seen in endometriosis might be an effect on PCO, thus have an impact later on endometriosis (42, 43). Metformin is an oral biguanid, which inhibits neoglucogenese in the liver and as a consequence decreases blood glucose levels. Metformin, with its unique therapeutic potential, is a soft inhibitor of the mitochondrial energy machinery, which is the main driving force of proliferation. Endometriosis has a lot of similarities with cancer development (44). Metformin is used recently in combination with chemotherapy for its unique antiproliferative effect in cancer treatment (45). Like cancer, endometriosis is caused by similar mechanisms like generation of reactive oxygen species (ROS), Warburg effect, stem cell differentiation and epithelial-mesenchymal transition (EMT). Retrograde menstruation can induce high levels of free iron in endometriotic cysts, which stimulates the production of reactive oxygen species (ROS) through the Fenton reaction and activated tissue-associated macrophages. This contributes to the increase of PGF2α into the peritoneal micro-environment, which is the cause of inflammation and favoring a proliferative process activated by cytokine activation (46, 47). At the same time pelvic pain arises. For many reasons described as anti-inflammatory, anti-angiogenetic and anti-proliferative effect, metformin may become the missing non-hormonal treatment for endometriosis patients.

### When Is Metformin an Interesting Choice?

Metformin could be utilized in endometriotic patients with contraindications to hormonal therapy, in women who desire pregnancy, in patients with side effects to medical therapy, or in patients resistant to other treatments.

Metformin could also be combined with hormonal treatment allowing a reduction of the dosage. Especially in young patients, early detection and treatment with tolerable side effects is important as it may prevent advanced disease. Additionally, Metformin has an indirect effect on estradiol, as it eases weight loss. Reduction of fat tissue reduces endogenous estradiol and consequently has a positive effect on endometriosis implants (17). Advanced stage endometriosis is observed more frequently in obese patients (48). Obesity is associated to hyperestrogenic rate. This study established an increased disease severity and reduced frequency of stage I endometriosis. It remains unclear what role body mass index has in the cause or effect of endometriosis.

Patients with endometriosis may have a beneficial effect when they are undergoing infertility treatment. A Finnish study of 320 women who received metformin (1,500–2,000 mg/day) or placebo for 3 months prior to fertility treatment, and for a further 9 months during treatment and up to 12 weeks of gestation, increase in pregnancy rate from 40.4 to 53.6% (OR 1.61, 95% CI 1.13–2.29), with obese women experiencing the greatest benefit. Furthermore, the live birth rate was increased in those who received metformin (41.9 vs. 28.8%; *P* = 0.014). Taking these results and those from further studies, obesity seems to have the greatest impact on the risk of miscarriage and metformin appears to reduce miscarriage in obese women (49).

## Conclusion

The studies investigating the effect of Metformin in endometriosis are promising. The published studies using *in vitro* and animal models illustrate that Metformin is implicated with the regression of endometriotic implants. However, there are still insufficient studies available on the effects of Metformin and endometriosis. Unfortunately, the studies are heterogeneous and clinical testing is sparse. For this reason, further investigation of the potential of metformin as an anti-endometriotic drug is needed. Prospective clinical studies are necessary to confirm the use of Metformin in the treatment of endometriosis.

## Data Availability Statement

The datasets presented in this article are not readily available. Requests to access the datasets should be directed to attila.major@outlook.

## Author Contributions

AM had the original idea. AM, ES-L, and TG conceived and drafted the original version of the article. AF, JB, and J-MA revised the article. All authors discussed and contributed to the final manuscript.

## Conflict of Interest

The authors declare that the research was conducted in the absence of any commercial or financial relationships that could be construed as a potential conflict of interest.
